# Design-based research on teacher facilitation practices for serious gaming in formal schooling

**DOI:** 10.1186/s41039-017-0056-6

**Published:** 2017-09-19

**Authors:** Morris Siu Yung Jong, Anmei Dong, Eric Luk

**Affiliations:** Department of Curriculum and Instruction / Centre for Learning Sciences and Technologies, The Chinese University of Hong Kong, Sha Tin, Hong Kong

**Keywords:** Game-based learning, Serious games, Teacher facilitation, Formal schooling

## Abstract

Serious gaming has been regarded as one of the important student-centric learning approaches in the coming decade. However, there has been a lack of in-depth discussion of the teacher role in the course of serious gaming when it is adopted in formal schooling. The study discussed in this paper is a piece of two-cycle design-based research, involving three teachers respectively from top, middle and bottom academic banding schools in Hong Kong and their Grade 11 classes in two consecutive school years (197 students in total). In the context of formal curriculum learning and teaching, we (researchers) collaborated with the teachers (practitioners) to investigate (design, enact, analyse and redesign) what and how they should do in order to optimise their students’ serious gaming process and advance the pedagogic effectiveness of serious gaming in different classroom settings.

## Introduction

Constructivist Online Game-Based Learning (COGBLe) has received a lot of attention from educators and researchers since the beginning of this decade (Bagley & Shaffer, 2015; Chee, 2016; Gee, 2013). The momentum of this learning and teaching initiative in school education can be reflected in the New Media Consortium’s Horizon Report K-12 Edition 2015 (Johnson et al. 2015) that has predicted there would be more and more adoptions of COGBLe in school education in the coming triennium.

Recent COGBLe studies can be divided into two main streams (Jong, Lee & Shang, 2013). One is about harnessing existing commercial off-the-shelf *recreational games* for instructional or pedagogical use (e.g., Gee, 2013; Keskitalo et al. 2011; Lan, 2015; Lin & Lan, 2015). The other is about *serious gaming*. It involves developing educational or training games which are implemented with sophisticated simulations and specific educative aims and contents (e.g., Arici & Barab, 2014; Iten & Petko, 2016; Tseleves et al. 2016; Tuzun & Ozdinc, 2016). Our work goes to the latter.

Constructivist education emphasises not only the active and self-directed role of learners (Papert, 1993) but also the vitality of teacher facilitation in the course of learning (e.g., Collins et al. 1989; Howland et al. 2012; Lave & Wenger, 1991; Tsai & Chai, 2012; Vygotsky, 1978). Regrettably, the prior scholarship of serious gaming has largely ignored the facilitation role of teachers in the pedagogic process (Jong, Lee & Shang, 2013; Berg Marklund & Alklind Taylor, 2015). Our work aims to address the research gap.

VISOLE (Virtual Interactive Student-Oriented Learning Environment) is a teacher-supported pedagogic framework that we earlier designed for integrating serious gaming into formal learning and teaching in school education (Cheung et al. 2008; Jong, Shang, Lee & Lee, 2010). The framework emphasises the importance of teacher facilitation components, scaffolding (Vygotsky, 1978) and debriefing (Crookall, 1992), respectively before, during and after the course of serious gaming.

The study discussed in this paper is a piece of design-based research (Design-based Research Collective, 2003; Mckenney & Reeves, 2012) with two research cycles in two consecutive school years. In the study, we collaboratively worked with teachers (practitioners) to improve and enhance the teacher facilitation components in VISOLE via implementing serious gaming in teaching a 6-week senior secondary Geography formal curricular module. The first-cycle research findings have been reported in our previous publications (Jong, 2015; Jong & Shang, 2015). This paper focuses on reporting on the results of the second-cycle research analysis that compared the pedagogic effectiveness of the optimised teacher facilitation practices with (i) the originally designed in VISOLE and (ii) the traditional textbook-based teaching, regarding the promotion of students’ knowledge acquisition.

We organise the rest of the paper as follows. The next section is a quick review of the related work. Then, we will delineate the research design in which we will recap parts of our prior work so that readers can gain a better understanding of the research background. Afterwards, we will present and discuss the findings, limitations and implications of the study. At the end of this paper, we will give our concluding remarks, as well as proposing the future work.

## Related work

### Serious gaming

Unlike traditional drill-and-practice mini-games for the purpose of sugaring the pills (Prensky, 2001), *serious games* are developed with state-of-art digital technology, designed and implemented with dedicated pedagogy, and embedded with specific educative content (Games & Squire, 2011). For example, in Shaffer et al.’s serious games (Bagley & Shaffer, 2015; Nash & Shaffer, 2013; Shaffer, 2009; Shaffer & Graesser, 2010), *distributed authentic professionalism* is the underpinning pedagogy. They believe that members of a profession should have a specific way of thinking and working, namely, *epistemic frame*. Hence, developing a person to be an “insider” of a profession is a matter of empowering him/her with that particular frame. *Urban Science* is one of the serious games developed by Shaffer’s group. In this game, players are required to role-play a staff member of an urban planning company that handles various land use issues in ecological areas. Via ongoing interactions with different game characters, the epistemic frame of ecologists will be infused into the players’ minds in a spontaneous fashion.

In fact, against the backdrop of the advocacy of constructivist education in the twenty-first century, the proposition of harnessing serious gaming in learning and teaching is raging (Johnson et al., 2015). However, evidence of its widespread adoption in school education still appears to be lacking (Chee, 2016). So far, most of the serious gaming studies and instances have been aimed at supporting informal learning outside the school contexts, or carrying out short-term learning experiments for testing educational hypotheses (Tobias et al. 2011). The developed serious games are not targeted on supporting formal subject-based curriculum learning and teaching in schools (Chee, 2016; Gee, 2013). Therefore, it is hard for school teachers to adopt serious gaming in practice (Jong, 2015; Jong & Shang, 2015).

As aforementioned in the introduction part of this paper, another major limitation in the scholarship of serious gaming is the ignorance of teacher facilitation in students’ game-based learning process. In fact, notwithstanding the emphasis of the active, self-directed and learner-centric role for students in various constructivist learning theories, teachers are always regarded as the major person to scaffold students to attain the educative goals in the course of learning (e.g., Collins et al. 1989; Howland et al. 2012; Lave & Wenger, 1991; Tsai & Chai, 2012; Vygotsky, 1978). Serious gaming should be no exception (Jong, Lee & Shang, 2013).

### Design-based research (DBR)

Researchers may not always be able to provide practitioners with desirable solutions to be applied in real-world contexts (Wang & Hannafin, 2005). Design is research; research is design (Cobb et al. 2003). Design-based research (hereinafter referred as DBR) aims to improve or enhance innovations through a collaborative effort among researchers and practitioners and via recursive research cycles of development and implementation (Design-based Research Collective, 2003). DBR situates applied work in authentic, naturalistic settings (Wang & Hannafin, 2005), with the aim of “more than understanding the happenings of one particular context, but also requires showing the relevance of the findings derived from the context of intervention(s) to other contexts” (Barab & Squire, 2004, p. 5).

In the domain of education, DBR is particularly useful for generating usable knowledge that sheds light on developing or revamping educational practices (Lagemann, 2002; Mckenney & Reeves, 2012). Researchers use this methodological approach to design interventions for tackling real-world problems taking place in education and then empirically implemented in authentic educational contexts (Mckenney & Reeves, 2012).

**Fig. 1:**
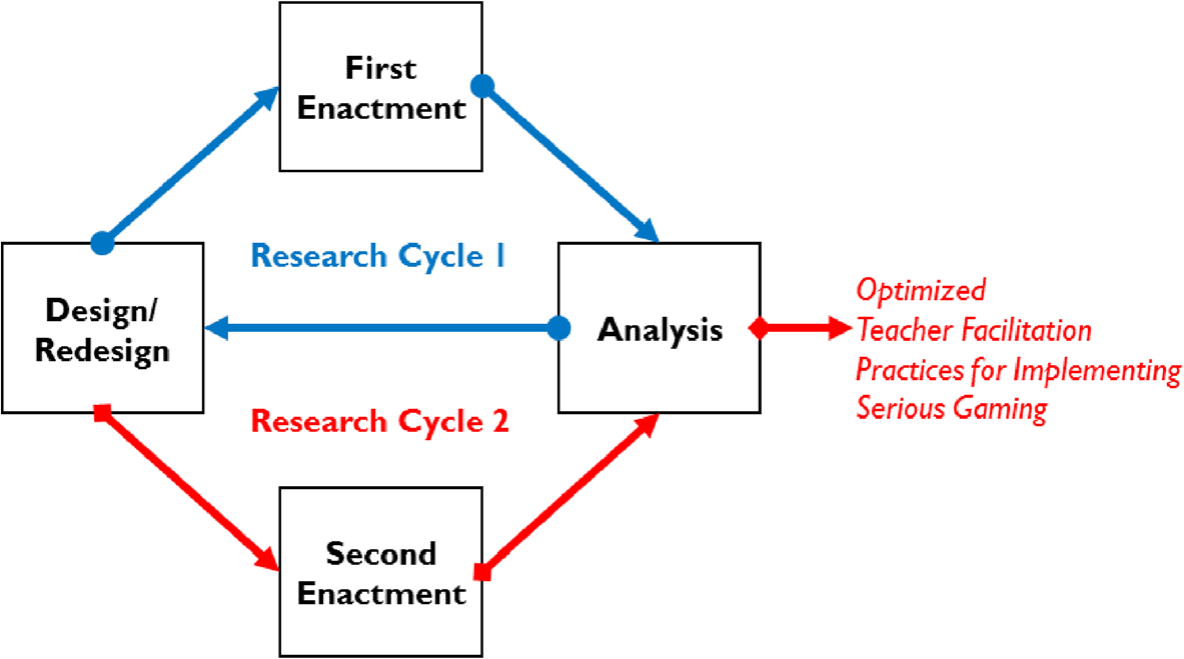
Two-cycle DBR design

A number of DBR paradigms have been proposed by various DBR researchers, while Design-based Research Collective’s (2003) which has been largely cited in many important DBR references (e.g., Barab & Squire 2004; Philips et al. 2012; Mckenney & Reeves, 2012;) is perhaps the most well-known one. It characterises the course of DBR by iterative research cycles of *design*, *enactment*, *analysis* and *redesign* (see Fig. 1). The “output(s)” of the previous research cycle will steer the focal investigation of the next research cycle.

## Methods

We employed Design-based Research Collective’s (2003) four-stage DBR methodological approach to achieving the aim of the present study, i.e*.*, to investigate what and how teachers should do in order to optimise students’ serious gaming process and advance the pedagogic effectiveness of serious gaming in different classroom settings. The study consisted of two research cycles, as illustrated in Fig. 1. As aforesaid in the introduction part of this paper, the first-cycle research findings have been reported in our previous publications (Jong, 2015; Jong & Shang, 2015). This paper focuses on reporting on the second-cycle research findings and comparing the pedagogic effectiveness of the optimised teacher facilitation practices with the originally designed in the first research cycle, in terms of promoting students’ knowledge acquisition. However, in order to give readers a better understanding of the research background, we will recap parts of our previous work in the following.

### Design

VISOLE (Virtual Interactive Student-Oriented Learning Environment) is a teacher-supported pedagogical framework that we initially designed for integrating serious gaming into formal learning and teaching in school education (Cheung et al. 2008; Jong, Shang, Lee & Lee, 2010). It emphasises the importance of teacher facilitation components, scaffolding (Vygotsky, 1978) and debriefing (Crookall, 1992), respectively before, during, and after the course of serious gaming. There are three phases in this framework. In Phase 1 (before the start of serious gaming), the teacher scaffolds students with initial abstract knowledge related to the subject matter that the serious game intends to cover. The pedagogic activities in Phases 2 and 3 are crossed together. Phase 2 engages students in serious gaming in which they will shape the development of the “virtual world” therein. All tasks in this “world” are authentic and open-ended in nature. Students should acquire new knowledge on their own from other learning resources (e.g., from the Internet) in order to accomplish the tasks. In addition, after the completion of each gaming round, they are required to write a short journal to reflect on what they have learned. In Phase 3, the teacher observes students’ gaming at the backend and extracts cases taking place in the game for debriefing students during and after the course of gaming.

Based on the VISOLE framework, we developed a 12-round online multiplayer serious game with respect to the Agriculture module in the Grade 11 Geography curriculum in Hong Kong (Cheung et al. 2008; Jong, Shang, Lee & Lee, 2010). In the game, each player acts as a farm manager running a farm. Each of them contends with other players (who are the managers of nearby farms in the virtual world) on the overall farm revenue. Therefore, they have to derive good operational acts in order to generate quality farm products. To support students in reflecting on their learning in Phase 2 of VISOLE, a blogging gadget is added to the game. After each round of gameplay, it will pop up to remind students to do reflective blogging. In addition, to facilitate teachers to conduct the debriefing work in Phase 3 of VISOLE, a web-based teacher console for retrieving students’ gaming data is integrated into the game system. Teachers can use the console to observe and capture students’ gameplay proceedings in the form of video playback.

In general, every week there are two lessons (about70 minutes each) dedicated for Geography in senior secondary education in Hong Kong. Using the traditional textbook-based approach, Geography teachers normally spend 6 weeks on teaching the Agriculture module. In our design, teachers will use the same time span to implement the VISOLE approach (with the game) in teaching the same module, as shown in Table 1 (Jong, 2015; Jong & Shang﻿, 2015).

**Table 1:** Implementation schedule of VISOLE with the game

Week	First lesson	Second lesson	Round(s) at home
1	Scaffolding lesson 1	Scaffolding lesson 2	/
2	Scaffolding lesson 3	Game-trial lesson	Round 1
3	Gaming lesson 1 (round 2)	Debriefing lesson 1	Round 3
4	Gaming lesson 2 (round 4)	Debriefing lesson 2	Rounds 5, 6
5	Gaming lesson 3 (round 7)	Debriefing lesson 3	Rounds 8, 9
6	Gaming lesson 4 (round 10)	Debriefing lesson 4	Rounds 11, 12

### Enactment

Secondary schools in Hong Kong are categorised into three academic bands based on their students’ academic achievement. Bands A, B and C are respectively the top, middle and bottom bandings. In this DBR, we collaborated with three Geography teachers respectively from the three different bandings to implement serious gaming in their teaching practice in two consecutive school years.

The 16 teachers who had participated in our early pilot study Jong, Shang, Lee & Lee, 2010) were put into the selection scope. Finally, three female teachers were selected because (i) they were teaching at different school bandings; (ii) their academic background and years of teaching experience were similar; (iii) the number of students in their Geography classes were comparable and (iv) the three schools were using the same Geography textbook (Jong, 2015). All of them possessed a bachelor’s degree in Geography, a postgraduate diploma in Geography education, and around 8 years of teaching experience. For writing convenience, hereinafter *School A and Teacher A*, *School B and Teacher B*, as well as *School C and Teacher C* are used to denote the corresponding schools and teachers at Band A, Band B, and Band C.

The first enactment (in the first research cycle) had been conducted in the school year of 2014 (Jong, 2015; Jong & Sha﻿ng, 2015). Adopting the VISOLE framework and the game, Teachers A, B and C implemented serious gaming to teach the Agriculture module in their schools. The total number of Grade 11 student participants in that enactment had been 99 (34 from School A, 32 from School B, and 33 from School C). The second enactment (in the second research cycle) was conducted in the school year of 2015. Adopting the optimised VISOLE framework and the revised game (see the sub-section of Redesign), Teachers A, B and C again implemented serious gaming to teach the Agriculture module in their schools. The total number of Grade 11 student participants in this enactment was 98 (35 from School A, 32 from School B, and 31 from School C).

During both the Enactment stages, we closely observed the implementation process in Schools A, B, and C. At the end of the Enactment stage in each cycle, in each school we conducted a knowledge test. The test, which was in the format of the conventional Hong Kong senior secondary public examination, contained 20 multiple-choice questions and two long questions (Jong, 2015). All questions were customised from the Agriculture-related questions in the past public examination papers. The perfect score was 50. To secure the validity, the test and marking scheme were scrutinised by a review panel composed of nine in-service Geography teachers possessing 10 to 16 years of teaching experience.

### Analysis

Apart from analysing the quantitative data collected via the knowledge test in each research cycle, we carried out in-depth interviews with the students to understand more about their experience in pursuing serious gaming right after the first and second Enactment stages. The further collected qualitative data were useful for triangulating the effectiveness of the teacher facilitation practices implemented and optimised in this DBR. We leveraged Maxwell’s (2012) qualitative analysis approach supplemented with Creswell’s (2015) thematic development technique of theme layering and interrelating to analyse the qualitative data (Jong, 2015; Jong & Shang, 2015).

### Redesign

The Redesign stage took place right after the end of the Analysis stage in the first research cycle (see Fig. 1). On the technical aspect, we newly integrated a number of non-player characters (NPCs) into the game as “virtual mentors” (e.g., Orchardist, Agricultural Biochemistry Specialist) to scaffold non-gamer students in the gaming process. The existence of these new NPCs in the game can be “by default” or “by injection” through the teacher console in a timely manner. In addition, the blogging gadget in the game was revamped to support both text-based and audio-based blogging. The revised game was pilot-tested before the second enactment (Jong, Shang, Tam, 2016).

On the pedagogic aspect, we discussed the findings obtained in the first research cycle with the three teachers. Then, we worked together to derive new interventions for optimising the existing facilitation practices in VISOLE in accordance with their schools settings. Table 2 shows a summary of the new interventions enacted on top of the original design of VISOLE at the Enactment stage in the second research cycle in each school. We will further discuss these optimised practices in the discussion part of this paper.

**Table 2:** Optimised teacher facilitation practices enacted in the second research cycle

	New interventions enacted at School A	New interventions enacted at School B	New interventions enacted at School C
Phase 1: scaffolding	• Equipping students with the gameplay knowledge via in-class practice and “flipped” video	• Leveraging gamer students’ proficiency to support non-gamer students in launching the game	• Video-recording the scaffolding lessons and putting the clips onto the web for students’ self-access
Phase 2: serious gaming and reflection	• Activating all “virtual mentors” for scaffolding students throughout the game • Assigning the gaming and blogging tasks as formal assignments for formative assessment	• Injecting the “virtual mentors” into the game in a just-in-time manner • Allowing students to do either text-based or audio-based blogging	• De-activating all “virtual mentors” in the game • Asking students to do audio-based blogging
Phase 3: debriefing	• Providing students with just-in-time encouragement for releasing their frustration and anxiety	• Facilitating group-based discussion in which students share their gaming and learning experience	• Inviting students to share their ongoing gaming strategies in front of the class

## Results

### Findings at School A

School A was a top academic banding school, and the student participants were high academic students. We received 33 completed test papers in the first research cycle (return rate = 97.06%) Jong, 2015) and received 33 completed test papers in the second research cycle (return rate = 94.29%). Table 3 shows the descriptive statistics of the students’ knowledge test results respectively in Research Cycles 1 and 2.

**Table 3:** Students’ knowledge test results obtained in Research Cycles 1 and 2 at School A

	Research Cycle 1 (Jong, 2015)	Research Cycle 2
Number of students	33	33
Mean	21.65	32.81
Standard deviation	7.01	7.98

An independent samples *t*-test on the knowledge test results indicated that the mean score in Research Cycle 2 (32.81) was significantly different from the mean score in Research Cycle 1 (21.65), *t*(64) = 5.95, *p* < 0.001). The Cohen’s *d* was 1.49. In other words, the optimised teacher facilitation practices and the revised game could significantly advance the pedagogic effectiveness of serious gaming in School A with a large effect size (Cohen, 1998), in terms of knowledge acquisition.

**Fig. 2:**
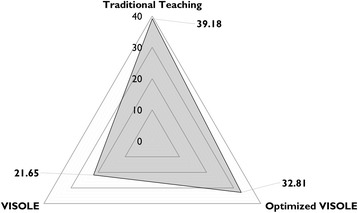
Overall comparison on knowledge test mean scores obtained at School A

Another independent samples *t*-test analysis was conducted to compare the knowledge test results of Research Cycle 2 and the control manipulation carried out during Recycle Cycle 1 at School A in which Teacher A had taught the control-group students (another class of 33 Grade 11 students in 2014) with the traditional textbook-based teaching approach (Jong, 2015). It indicated that the mean score in Research Cycle 2 (32.81) was still significantly lower than the mean score in the control manipulation (39.18), *t*(64) = 3.48, *p* < 0.001). The Cohen’s *d* was −0.86. In the individual interviews with the students after the Enactment stage in the second cycle, we did find further clues to explain the overall findings at School A (Fig. 2 as an overall illustration). The following are some related excerpts of the interviews.

- Andrew (pseudonym):
  This is the first time I have played such a complicated game …… at the beginning, I thought that I would give up playing it. The operation and gaming style of this game were totally new to me. I appreciate the effort that the teacher made to help us learn how to launch the game. I deem that the in-class practice before starting the game was important. The video produced by the teacher was very useful too. In the course of gaming, I did re-watch the video a few times.

- Andy (pseudonym):
  Honestly, I am not into online gaming although a lot of my friends love gaming very much …… My academic performance is always the first priority in my school life. If the teacher did not assign the gaming and blogging tasks as formal assignments, I would not be so enthusiastic about finishing the tasks ……. However, after playing some rounds, I started to understand why the teacher introduce this game to us. The game offered us opportunities to apply our gained knowledge in a near real-world context of Agriculture … as you know, Hong Kong has no farming industry …… I think I am still not good at game-based learning, Haha … I am more confident of learning in the traditional teaching setting.

- Angela (pseudonym)
  My bad ongoing gaming results in the first three rounds frustrated me a lot, but many thanks for the encouragement given by the teacher. She reminded me about the primary aim of this activity was learning, not gaming. My learning attainment could not only be reflected from my gaming score but also from my reflective blogging …… the process of blogging helped me clear up many misconceptions and reinforced my knowledge about Agriculture.

- Angie (pseudonym):
  I had no experience in playing such a complex game before, but you know, finally I won the game, Haha …… I should give credit to the NPCs “who” offer me a lot of guidance in the game. I like this learning approach. I think all of us can do even better (in both gaming and learning) if we are provided with more opportunities for practising game-based learning.

### Findings at School B

School B was a middle academic banding school, and the student participants were moderate academic students. We received 31 completed test papers in the first research cycle (return rate = 96.86%) Jong, 2015 ) and received 30 completed test papers in the second research cycle (return rate = 93.75%). Table 4 shows the descriptive statistics of the students’ knowledge test results respectively in Research Cycles 1 and 2.

**Table 4:** Students’ knowledge test results obtained in Research Cycles 1 and 2 at School B

	Research Cycle 1 (Jong, 2015)	Research Cycle 2
Number of students	31	30
Mean	28.11	35.60
Standard deviation	7.45	8.01

An independent samples *t*-test on the knowledge test results indicated that the mean score in Research Cycle 2 (35.60) was significantly different from the mean score in Research Cycle 1 (28.11), *t*(59) = 3.74, *p* < 0.001). The Cohen’s *d* was 0.97. In other words, the optimised teacher facilitation practices and the revised game could significantly advance the pedagogic effectiveness of serious gaming in School B with a large effect size (Cohen, 1998) in terms of knowledge acquisition.

**Fig. 3:**
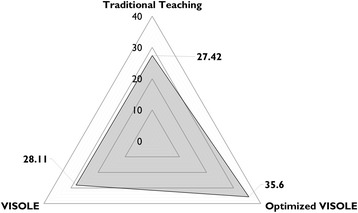
Overall comparison on knowledge test mean scores obtained at School B

Another independent samples *t*-test analysis was conducted to compare the knowledge test results of Research Cycle 2 and the control manipulation carried out during Recycle Cycle 1 at School B in which Teacher B had taught the control-group students (another class of 32 Grade 11 students in 2014) with the traditional textbook-based teaching approach (Jong, 2015). It indicated that the mean score in Research Cycle 2 (35.60) was also significantly higher than the mean score in the control manipulation (27.42), *t*(60) = 4.18, *p* < .001). The Cohen’s *d* was 1.08. In the individual interviews with the students after the Enactment stage in the second cycle, we did find further clues to explain the overall findings at School B (Fig. 3 as an overall illustration). The following are some related excerpts of the interviews.

- Benjie (pseudonym):
  This game is very sophisticated, unlike the mobile mini-games, Angry Birds™, Draw Something™, etc. that I played before. The gamer classmates helped me a lot. Without their support, I think I would give up playing it at the very beginning.

- Benny (pseudonym):
  I’ve later learned that the NPCs were injected by the teacher after she had observed that I might need help in the game. Through interacting with the NPCs, I gained more agricultural tips to run my farm. My gaming strategies became more sophisticated in the latter rounds.

- Bethany (pseudonym):
  I like this learning-by-gaming approach very much. I was fully engaged in this activity …… I don’t like writing, so I opted for doing audio-based blogging. I talked a lot via the blogging gadget. You can check it out …… Every sound bite is my in-depth learning reflection.

- Betty (pseudonym):
  The atmosphere of the debriefing lessons was very engaging. The teacher put gamers and non-gamers into the same group. For example in my group, we shared our own experience in the game and gave suggestions to one another about what gaming strategies we could adopt in the next few rounds in order to maximise the revenue.

### Findings at School C

School C was a bottom academic banding school, and the student participants were low academic students. We received 33 completed test papers in the first research cycle (return rate = 100%) (Jong, 2015) and received 31 completed test papers in the second research cycle (return rate = 100%). Table 5 shows the descriptive statistics of the students’ knowledge test results respectively in Research Cycles 1 and 2.

**Table 5:** Students’ knowledge test results obtained in Research Cycles 1 and 2 at School C

	Research Cycle 1 (Jong, 2015)	Research Cycle 2
Number of students	33	31
Mean	27.11	32.09
Standard deviation	7.13	7.88

An independent samples *t*-test on the knowledge test results indicated that the mean score in Research Cycle 2 (32.09) was significantly different from the mean score in Research Cycle 1 (27.11), *t*(62) = 2.66, *p* < 0.01). The Cohen’s d was 0.66. In other words, the optimised teacher facilitation practices and the revised game could significantly advance the pedagogic effectiveness of serious gaming in School C with a medium-large effect size (Cohen, 1998), in terms of knowledge acquisition.

**Fig. 4:**
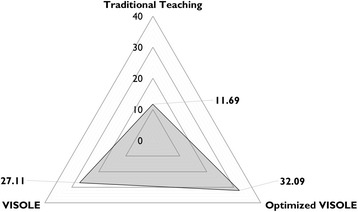
Overall comparison on knowledge test mean scores obtained at School C

Another independent samples *t*-test analysis was conducted to compare the knowledge test results of Research Cycle 2 and the control manipulation carried out during Recycle Cycle 1 at School C in which Teacher C had taught the control-group students (another class of 32 Grade 11 students in 2014) with the traditional textbook-based teaching approach (Jong, 2015). It indicated that the mean score in Research Cycle 2 (32.09) was again significantly higher than the mean score in the control manipulation (11.69), *t*(61) = 10.78, *p* < .0001). The Cohen’s *d* was 2.78. In the individual interviews with the students after the Enactment stage in the second cycle, we did find further clues to explain the overall findings at School C (Fig. 4 as an overall illustration). The following are some related excerpts of the interviews.

- Charles (pseudonym):
  I am a slow learner. Usually, it is not easy for me to grasp and understand the content taught inside the traditional classroom. However, this time all the (scaffolding) lessons were video-recorded and put on the web. I could access the video clips again and again … I did watch the clips several times before starting the game.

- Charlotte (pseudonym):
  I have learned from the teacher that there can be “virtual mentors” in the game for guiding players to accomplish the gaming tasks, right? It was so wise that the teacher turned off this function; otherwise, it would make the game boring. You know, gamers always want to explore the game mechanic by themselves, instead of being told or led by others.

- Charmaine (pseudonym):
  The idea of audio-based blogging is great. In these few weeks, I played every round and audio-recorded every piece of blogging. I had never been so responsible for my learning …… I hate writing. If the teacher requested me to text the blog, I would not do it for sure.

- Chris (pseudonym):
  There are a lot of game experts in this class. Of course, I am one of them, Haha. I did enjoy sharing my gaming tips with my classmates. In fact, I learned from others' sharing too …… When I was preparing the sharing, I have to deeply reflect on the scenarios taking place in my farm during the last few rounds. The reflective process transformed my gaming experience into my learning experience.

## Discussion

Collaborating with the three teachers and harnessing the pedagogic principles of scaffolding (Vygotsky, 1978) and debriefing (Crookall, 1992), we designed, enacted, analysed and redesigned specific teacher facilitation practices for implementing serious gaming in the context of formal curriculum learning teaching in different classroom settings.

At School A (a top academic banding school with high academic students), we found that there was significant advancement with a large effect size on the pedagogic effectiveness of the optimised VISOLE approach (see Table 2, the second column) in comparison with the original VISOLE approach’s. In the optimised approach, before launching the game, Teacher A made an effort on equipping the students with the gameplay knowledge via additional in-class practice and self-created gameplay instructional video. It effectively reduced, in Sweller et al.’s (2011) terms, “extraneous cognitive load” in serious gaming among the students who lacked prior complex gaming experience. Teacher A also set up extra scaffolds inside the game, i.e*.*, the “virtual mentors” (in Sweller et al.’s terms, “germane resources”) to further support the students throughout the gaming process. This positive pedagogic effect did align with what we had observed in our earlier pilot study on the revised game with non-gamer learners (Jong, Shang & Tam, 2016). Moreover, to provide a “valid reason” of participation for these “achieving learners” (Biggs & Moore, 1993) whose learning motive usually pivots on “getting higher scores,” assigning the game-based learning tasks as formal assignments became an important act in the setting. According to Koster (2005), it is inevitable that learners who are novices to online gaming will encounter frustration and anxiety in the course of serious gaming. Therefore, Teacher A kept closely monitoring the students’ emerging emotion via observing their blogs and offered them timely encouragement for releasing their negative emotion. Yet, the further comparison between the traditional textbook-based teaching approach and the optimised VISOLE approach indicated that the pedagogic effectiveness of the former was still significantly higher than the latter. However, when comparing with the results obtained in the first research cycles, i.e*.*, the traditional approach vs. the original VISOLE approach (Jong, 2015), we found that the difference diminished considerably (see the illustration in Fig. 2). Echoing the argument made by Angie (see “Findings at School A” in the previous section), we are interested in further investigating whether the pedagogic effect of serious gaming with the optimised VISOLE approach on these high academic students will increase if they are provided with more opportunities for practising “learning through gaming.”

At School B (a middle academic banding school with moderate academic students), in the first research cycle, we had found that there had been no significance difference between the pedagogic effectiveness of the original VISOLE approach and the traditional textbook-based teaching approach (Jong, 2015). Nevertheless, in the second research cycle, we found that there was significant advancement with a large effect size on the pedagogic effectiveness of the optimised VISOLE approach (see Table 2, the third column) in comparison with both the original VISOLE approach and the traditional textbook-based teaching approach, as illustrated in Fig. 3. The classes in School B were composed of gamers and non-gamers. In the optimised approach, Teacher B leveraged the gamers’ gameplay proficiency to support the non-gamers. She divided the class into a number of small groups. Each group was composed of both gamers and non-gamers to pursue collaborative discussion (Scardamalia & Bereiter, 2006). During the game-trial and debriefing lessons, the gamers in each group played the game-tutor role to share with the non-gamers about how to solve the difficulties encountered in the game. Indeed, not only the non-gamers but also the gamers benefited from this pedagogic manipulation. It is because “teaching others” is the best way for knowledge retention (Dale, 1969). According to Csikszentmihalyi (1975), when a person is working on a task, if the task is far below the one’s ability, it will induce boredom; on the contrary, if the task is far above the one’s ability, it will induce anxiety. Either boredom or anxiety may cause the person to escape from that task; in fact, we did witness this undesirable phenomenon in the first research cycle at School B (Jong & Shang, 2015). Considering that the class was a mixture of the gamers and non-gamers, instead of activating the “virtual mentors” in the game by default, Teacher B injected these scaffolds into the farms of the students who needed extra support in a just-in-time manner. Moreover, learners’ autonomy is regarded as a vital element in learner-centric environments (Niemiec & Ryan, 2009). Thus, offering the students flexibility to do either text-based or audio-based blogging was also an important intervention enacted at School B.

At School C (a bottom academic banding school with low academic students), we found that there was significant advancement respectively with a medium-large effect size and a large effect size on the pedagogic effectiveness of the optimised VISOLE approach (see Table 2, the fourth column) in comparison with the original VISOLE approach and the traditional textbook-based teaching approach, as illustrated in Fig. 4. In the optimised approach, before launching the game, Teacher C reinforced the abstract knowledge scaffolding part of VISOLE via a “flipped learning” strategy (Bergmann & Sams, 2014) which has been observed to be effective on low academic achievers in some recent studies (e.g., Baepler et al. 2014; Sahin et al. 2015; Zummo & Brown, 2016). As aforementioned, boredom may cause a person to escape from the task that he/she is pursuing (Csikszentmihalyi, 1975). Echoing the argument made by Charlotte (see “Findings at School C” in the previous section), de-activating the “virtual mentor” in the game was another vital practice enacted at School C because this class was composed of many experienced gamers. Motivating learners by making them perceive that they are competent to accomplish the tasks in the course of learning is always vital (Niemiec & Ryan, 2009). Echoing the argument made by Charmaine (see “Findings at School C” in the previous section), it was a wise act that Teacher C asked the students to do audio-based blogging rather than text-based blogging. Besides, the best way for knowledge retention is “teaching others” (Dale, 1969). As observed, the students did benefit from taking turns to share their game-based learning experience with their classmates in the debriefing lessons. It helped them transform their gaming experience into their learning experience.

## Limitations

Serious gaming is still a novel pedagogic idea to most teachers in Hong Kong. In fact, the three teacher participants in this DBR should be more or less interested and positive about serious gaming. Otherwise, they would not be willing to take the risk to put this educational innovation into real practice in their schools. If these teachers were replaced by randomly selected ones, we deem that the findings discussed in this paper would not be simply replicated.

Another possible limitation is the Hawthorne effect (McBride, 2015) on the student participants. It refers to the process where human research subjects change their behaviour because they are being investigated. In this DBR, the novelty of adopting a new game-based learning approach in formal schooling might lead to the promotion of the students’ learning achievement just in a temporary manner. Can the positive effect sustain? This should be a piece of further research.

## Implications

The integration of serious gaming into formal schooling and the importance of teacher facilitation in the process of game-based learning have received little attention in the field of COGBLe (Jong, Lee & Shang, 2013; Berg Marklund & Alklind Taylor, 2015). Our work alerts researchers to the gaps, as well as trying to address the shortfalls. Specifically, we aim to answer two important questions—(i) what and (ii) how schoolteachers could do when implementing serious gaming in the context of formal curriculum learning and teaching. It contributes to the development of teacher facilitation practices for implementing serious gaming and advances the knowledge of harnessing serious gaming in school education. The research findings provide game-based learning researchers and developers with new insights into creating serious games for formal schooling, as well as framing pedagogic strategies for implementing their games in different school settings. The findings also shed light for teacher educators (of both pre-service teacher education and in-service teacher professional development) with a pragmatic reference and a real example for designing pedagogic training that prepares teachers to adopt serious gaming in school education.

## Conclusions

COGBLe research has largely focused on game designs, as well as the relationship between gaming and learning outcomes such as promoted motivation, deepened engagement, and acquired knowledge and skills (Berg Marklund & Alklind Taylor, 2015). Different from the foci of the mainstream studies in the field, we explore what and how teachers should do in order to optimise students’ learning process in COGBLe in the context of formal schooling. In this two-cycle DBR, working closely with the three teachers and leveraging the pedagogic principles of scaffolding (Vygotsky, 1978) and debriefing (Crookall, 1992), we achieved the aim of designing, enacting, analysing and redesigning effective teacher facilitation practices for advancing the pedagogic effectiveness of serious gaming in different classroom settings.

In this study, the strategy of using “virtual mentors” (NPCs) to scaffold non-gamer learners was proved to be effective. It is worthy of further technical research effort on making the NPCs more “intelligent” with artificial intelligence (AI) technology so that they will timely appear in the game at critical moments, rather than manually injected by teachers. As aforesaid, the Hawthorne effect (McBride, 2015) is a possible limitation of this DBR. There is a need for further investigation on the substantiality of the pedagogic effect of the formulated teacher facilitation practices on the students who participated in the second research cycle when they learn with the optimised VISOLE approach for the second time and third time. Hence, it will involve developing additional VISOLE games for other topics in the Geography curriculum or even in other subjects. Last but not the least, the idea of harnessing serious gaming in formal schooling is still novel in Hong Kong (Jong, 2016). It is important to formulate effective strategies to (i) introduce serious gaming into pre-service and in-service teacher training programmes and (ii) address teachers’ concerns about adopting serious gaming in practice with articulated support.
